# Renal Biomarkers and Novel Therapies in Pediatric Nephrology: From Chronic Kidney Disease to Renal Transplantation

**DOI:** 10.3390/jcm12113810

**Published:** 2023-06-01

**Authors:** Valeria Chirico, Roberto Chimenz

**Affiliations:** Pediatric Nephrology and Dialysis Unit, University Hospital “G. Martino”, 98124 Messina, Italy

Chronic kidney disease (CKD), a growing problem with an estimated prevalence of 74.7 cases per million children, induces high morbidity, mortality, and poorer quality of life [1,2].

Congenital anomalies of the kidney and urinary tract (CAKUT) represent 50% of the causes of CKD, followed by inherited nephropathies and glomerulonephritis [1]. Furthermore, in all these patients, blood hypertension is an independent risk factor for renal and cardiovascular diseases [3]. However, whether blood pressure monitoring represents a reliable tool is debated, highlighting that recent research has not identified a correlation between nocturnal blood pressure dipping and CKD progression in mild-to-moderate CKD pediatric patients [4].

Prompt diagnosis and proper management are essential, considering that late referral represents a not negligible issue, as observed in developing countries where end-stage renal disease (ESRD) is the first manifestation of kidney damage in more than half of children [5]. A precocious diagnosis and the prevention of renal disease progression are targets for inherited kidney diseases [6]. Proteinuria, hematuria, or extra-renal symptoms often occur in the late stages, whereas subclinical phases are not detected during childhood due to the lack of specific symptoms, signs, or biomarkers [7,8].

Next-generation sequencing accelerated the etiological diagnosis of CAKUT and inherited nephropathies with an accurate diagnosis and prognostic information, achieving a personalization of therapy and analyses of the risks related to the diseases [9].

Improvements have been also achieved in the pharmacological field in recent years. Primary hyperoxaluria (PH1), an inherited disorder involving glyoxylate metabolism and causing significant increases in hepatic oxalate production, leads to recurrent kidney stones, nephrocalcinosis, and kidney failure. It is a potentially life-threatening systemic disease, but the diagnosis is often delayed or missed; CKD is still the most common presentation [10].

Before 2020, the treatment of PH1 was only based on supportive measures, such as intensive water intake, vitamin B6, and oral crystallization inhibitors, while by November 2020, the Food and Drug Administration and the European Medicines Agency approved the use of lumasiran, improving the kidney outcome, especially if administered early [11].

Similarly, nonspecific clinical phenotypes characterized the Fabry disease in childhood, with a high rate of misdiagnosis and damage in adulthood. Only in-depth screening programs of high-risk groups and the creation of networks between different specialties could improve diagnosis and therapy management [12,13]. In this context, the long-term effect of enzyme replacement therapy may delay the progression of the disease in Fabry patients [14].

These are examples of rare diseases with an underestimated incidence due to a poor diagnosis but with a great potential to change the natural history of the disease, based on dialysis and transplantation, by newly available therapies.

In adulthood, sodium-glucose cotransporter-2 inhibitors (SGLT-2), also named gliflozins, can delay the progressive decline of kidney function behind preexisting type 2 diabetes mellitus. For diabetic pediatric patients, dapagliflozin can reduce insulin requirements, lowering HbA1c and body mass index [15]. Ongoing trials analyzing different outcomes of the pediatric population treated with gliflozin will provide future recommendations for this SGLT2 inhibitor, as observed in adulthood [16].

Children with acute kidney injury (AKI) can develop chronic kidney damage. Additionally, in children, the search for sensitive and specific biomarkers of AKI is still a challenge. Behind the complexity of AKI, which could be related to several comorbidities, ongoing tubular development, greater renal reserve, and superior renal regenerative potential compared with adults determine further difficulties in the diagnostic and prognostic fields [17]. The incidence and outcome of AKI remain largely unknown. Hospitalized children with AKI have not been extensively studied for their clinical outcomes, even if higher odds of mortality, longer duration of hospital stay, increased requirement of mechanical ventilation, and increased costs have been related to AKI [18].

Diabetic ketoacidosis is often associated with severe AKI, and besides volume depletion, hyperglycemia can induce tubular injury and kidney inflammation associated with low serum bicarbonate and low sodium [19]. Moreover, one-third of non-dialyzed Shiga-toxin Escherichia Coli hemolytic uremic syndrome (STEC-HUS) patients evolved to CKD after a median time of 5 years, with CKD being assessed even after 15 years, reinforcing the concept that all AKI patients should be followed until adulthood [20].

In these contexts, the measurement of the glomerular filtration rate (GFR) and its adequacy play central roles in the nephrology and pediatric field, whereas the measurements of GFR with inulin clearance in children could be only applied to research fields. As a reference of normal values, several equations were designed to estimate GFR and are the most widely used in children [21,22].

Estimated GFR (eGFR) is based on a calculation using an endogenous marker, such as creatinine or cystatin C. Due to its reliance on serum creatinine, it is quite inaccurate in children with a low muscle mass and is significantly affected by protein intake, height, gender, body mass, and hydration status. Moreover, serum creatinine is not a reliable marker to detect AKI as it rises when about 50% of the glomerular function is lost. The advantage of measuring Cystatin C is that age, gender, or body habitus do not influence it [23].

Albuminuria, a classic biomarker of kidney damage, precedes any decline in eGFR, but tubulo-interstitial diseases do not influence its levels. Thus, pediatric nephrologists require more sensitive and specific biomarkers of kidney damage as these would allow an earlier prediction of CKD. The early anticipation of the late stages of CKD, slowing down disease progression, could achieve better renal outcomes.

In acute conditions, a recent study assessed the predictive accuracy of urine Cystatin C at the cut-off of 1.26 mg/g urine creatinine to identify a greater risk of AKI in critically ill children at the age of more than one month [24].

In the last decade, several renal biomarkers were evaluated as a potential “troponin” of the kidney. While electrocardiogram alterations and troponin levels help cardiologists to define acute heart damage accurately and precociously, nephrologists base all their clinical activities and decisions on serum creatinine or urinary output variations, which are neither sensitive nor specific for AKI. Future markers should identify the stage of injury, anticipating a late increase in creatinine levels, occurring when the renal function already begins to decline. In particular, kidney injury molecule-1 and neutrophil gelatinase-associated lipocalin were widely tested, but despite their advantages over traditional parameters, such as albuminuria, they still require validation before they can be applied in clinical practice.

All these discussed points represent unmet needs, which justify effective efforts for children with kidney disease, hence the need for pediatric nephrology clinics and updated recommendations to improve the management of renal diseases [25].

Current structures are not adequate for meeting the demands of pediatric CKD, as well as dedicated outpatient clinic management for children who suffered from AKI and with a high risk of renal disease progression.

Managing a child or an adolescent with ESRD as the first expression of the disease, due to misdiagnosis or a late referral, represents a clear defeat of the entire system.

Children on regular hemodialysis (HD) have increased cardiovascular mortality compared to healthy subjects, contributing to nearly 40% of all-cause mortality [26].

Notwithstanding these data, also in the HD field, technological developments have occurred in recent last years, allowing the application of different sensors and software, creating personalized biofeedback systems with real-time and online monitoring devices, with sophisticated machines equipped with internal computers capable of hosting complex controllers of multi-input and multi-output types [27]. Moreover, modifications of the composition of hemodialysis membranes have improved their biocompatibility and patients’ quality of life, as observed by dialyzer membranes coated with bioactive compounds [28] or by replacing the minimal content of acetate with citrate in the dialysate [29].

A cohort of HD pediatric patients was treated with citrate-based dialysate, revealing a low percentage of thrombotic episodes, catheter or circuit loss, and mild and asymptomatic hypocalcemia [30].

In peritoneal dialysis (PD), biocompatibility has been improved in recent years, acting on the quality of the solutions, achieving a neutral pH, low glucose degradation product solutions, and icodextrin, influencing favorably some patient-level outcomes [31].

However, several factors affect the choice of dialysis modality to manage a specific patient. In the United States, PD is the most utilized dialysis modality (~55%) compared to HD (~44%). In the choice of dialysis technique, age is a key factor. Maintenance dialysis treatment using PD is preferred in patients <5 years of age (85%), whereas HD is common (50%) in children ≥13 years of age [32]. Moreover, PD is preferred for the management of AKI and in patients with hemodynamic instability [33].

Furthermore, the development of new continuous kidney replacement therapy machines helps nephrologists manage neonatal or small children with AKI. New monitors have smaller circuits that reduce extracorporeal volume and limit the need for blood priming, have more precise control systems that minimize machine errors, provide smoother flow rate adjustment, and allow the use of smaller-sized catheters, with advantages including fluid removal and better depuration [34].

However, the ideal therapy for dialyzed children is a transplant. Survival rates with kidney transplantation are better than HD or PD, even if cardiovascular complications, infections, and cancers are principal causes of late mortality for all patients [35].
